# Deep Eutectic Solvents as Promising Green Solvents in Dispersive Liquid–Liquid Microextraction Based on Solidification of Floating Organic Droplet: Recent Applications, Challenges and Future Perspectives

**DOI:** 10.3390/molecules26237406

**Published:** 2021-12-06

**Authors:** Asmaa Kamal El-Deen, Kuniyoshi Shimizu

**Affiliations:** 1 Department of Agro-Environmental Sciences, Faculty of Agriculture, Kyushu University, Fukuoka 819-0395, Japan; asmaakamal91@mans.edu.eg; 2 Department of Pharmaceutical Analytical Chemistry, Faculty of Pharmacy, Mansoura University, Mansoura 35516, Egypt

**Keywords:** deep eutectic solvent, DLLME-SFOD, green solvent, sample preparation

## Abstract

Deep eutectic solvents (DESs) have recently attracted attention as a promising green alternative to conventional hazardous solvents by virtue of their simple preparation, low cost, and biodegradability. Even though the application of DESs in analytical chemistry is still in its early stages, the number of publications on this topic is growing. Analytical procedures applying dispersive liquid–liquid microextraction based on the solidification of floating organic droplets (DLLME-SFOD) are among the more appealing approaches where DESs have been found to be applicable. Herein, we provide a summary of the articles that are concerned with the application of DESs in the DLLME-SFOD of target analytes from diverse samples to provide up-to-date knowledge in this area. In addition, the major variables influencing enrichment efficiency and the microextraction mechanism are fully investigated and explained. Finally, the challenges and future perspectives of applying DESs in DLLME-SFOD are thoroughly discussed and are critically analyzed.

## 1. Introduction

Despite unimpeachable improvements in analytical instrumentation, sample preparation still represents the major bottleneck and a greater time-consuming step in developing an analytical method [1]. It is not only necessary for analyte dissolution but also for sample homogenization, extraction, clean up, and preconcentration [2]. As such, it is considered the key to an accurate analysis. Upon considering the impact of analytical procedures to the environment, sample preparation was found to be the most challenging step regarding the green parameters of a method. It consumes large amount of toxic conventional solvents [3]. This can be overcome by different strategies: (i) miniaturizing the analytical scale through reducing chemical consumption and thus reducing waste generation [4] or (ii) replacing hazardous solvents with more sustainable and green alternatives [5,6]. Ideally, “solvent-free” sample preparation would be developed; however, this is still conceptual. Consequently, finding alternative solvents is essential [4,7,8]. In 2006, Rezaee et al. introduced dispersive liquid–liquid microextraction (DLLME) as an important miniaturized sample pre-treatment technique [9]. In DLLME, the spread of an extractant solvent in an aqueous sample is achieved by means of a dispersive solvent [10]. However, the majority of the solvents that are used in this technique are highly toxic, such as halogenated hydrocarbons [11]. Accordingly, Zanjani et al. reported a new liquid–liquid microextraction technique based on solidified floating organic droplets [12]. After extraction, the sample solution is transferred to an ice bath, allowing the floating organic droplets to solidify. The solidified floating organic droplets are then transferred and are allowed to melt before being used for analysis. This technique has the merits of being simple and uses less hazardous organic solvents than DLLME does. However, the spherical geometry of the floating droplet decreases the interfacial surface, thus reducing the extraction efficiency [13]. This challenge might be overcome through the use of a dispersive solvent that could be used to disperse the organic droplets throughout the sample solution in the form of tiny droplets [14,15]. This, in turn, significantly improves the interfacial area and thus enhances the extraction efficiency. This technique is known as DLLME-SFOD. Since its development, DLLME-SFOD has found applications as the preconcentration for different analytes from various matrices [16,17,18]. Most of the solvents that are used in DLLME-SFOD have lower toxicity than the ones that are conventionally used in DLLME [13]. However, the search for more sustainable, green, biodegradable, and cheap solvents for DLLME-SFOD has begun [19]. Among the green solvents, great attention has been paid to deep eutectic solvents (DESs) in various fields of study over the past two decades after a study by Abbott et al. [20] noted an abnormally deep melting point depression in the eutectic composition of certain hydrogen bond donors (HBDs) and acceptors (HBAs).

Even though the application of DESs in analytical chemistry is still in its early stages, the number of publications on this topic is growing. Analytical procedures applying DLLME-SFOD are among the most appealing approaches in which DESs have been found to be applicable. Herein, we will provide a summary of the articles that are concerned with application of DESs in the DLLME-SFOD of target analytes from diverse samples to provide up-to-date knowledge in this area.

## 2. Dispersive Liquid–Liquid Microextraction Based on Solidification of Floating Organic Droplet (DLLME-SFOD)

DLLME-SFOD is based on a ternary component solvent system. Briefly, a mixture that is an appropriate composition for both extraction and dispersion is rapidly injected into an aqueous sample [21] to form a cloudy solution. This increases the surface area between the fine droplets of the extracting solvent and the aqueous sample and thus facilitates the quick transfer of analytes from the sample solution into the extraction phase [22]. Subsequently, upon centrifugation, the two phases are separated, and the floated organic phase is then allowed to solidify in an ice bath before further collected by a spatula and is allowed to melt to before it is subjected to analysis [12]. DLLME-SFOD has become a very popular microextraction technique. It has the advantages of a high extraction efficiency, increased enrichment factors (EF), a low required volume of the extraction solvent compared to other LLE techniques, enhanced extraction kinetics, and rapid equilibrium state attainment. Furthermore, there is no carry-over effect in DLLME-SFOD due to the utilization of a new droplet every time. Finally, no specialized equipment or instruments are required for DLMME-SFOD (except for the centrifuge), which makes this method easy to apply. However, DLLME-SFOD has some limitations. Most importantly, the number of organic solvents that fulfill the extraction solvent criteria is limited. Therefore, the search for new solvents that are applicable for DLLME-SFOD has grown. Among them, DESs have attracted attention as being a promising new generation of green solvents.

## 3. Deep Eutectic Solvents (DESs)

DESs are obtained by combining a mixture of two or several components (hydrogen bond acceptor (HBA), and hydrogen bond donor (HBD)) by means of hydrogen bond interaction [23]. The term “eutectic” is derived from the Greek word for low melting, as they have a lower melting point than their own components [20]. The charge delocalization that occurs through hydrogen bonding between HBD and HBA is responsible for the decrease in the melting point of the mixture relative to the melting points of the individual components [24].

DESs have created a tremendous revolution in many fields of chemistry, especially DLLME-SFOD, as evidenced by the massive number of articles that have been published on the subject. Because of the achievements that have been encouraged by their application in several scientific domains, DESs are regarded as being the next generation of solvents. One of the main merits of DESs is their easy preparation, which can be achieved by simply mixing the components with gentle heating, which explains their widespread use [25]. They also have the advantages of minimal toxicity, great biodegradability, and lower environmental impact compared to other solvents; a low eutectic point; and particular polarity, surface tension, and thermal stability [26]. Although most reports have described DESs as being non-toxic and biodegradable, others state that more investigation is required on this matter since the toxicity of DESs varies depending on their components. Therefore, the cautious handling of the terms non-toxicity and biodegradability must be considered. However, despite everything, DESs are believed to be more effective and environmentally friendly solvents than other traditional ones.

On the other hand, the high viscosity and density of DESs make their application problematic compared to other solvents [27].Therefore, temperatures that are higher than ambient [28], or additional help are typically required. However, the physicochemical properties of DESs can be tailored through the selection of proper HBD and HBA and their molar ratio or with the addition of water.

**Figure 1:**
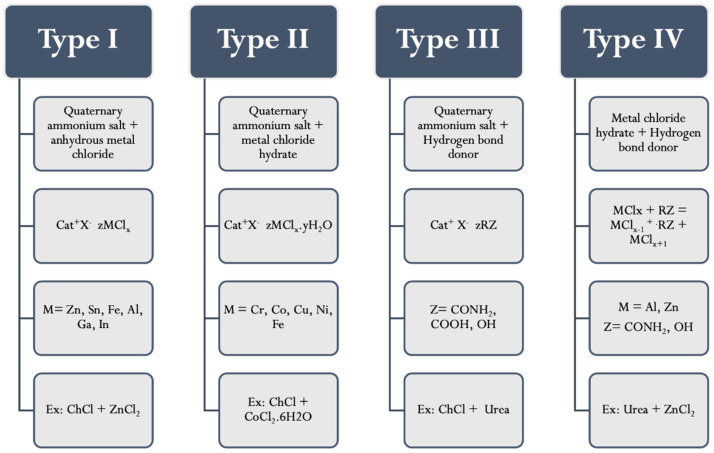
Types of deep eutectic solvents.

DESs are classified into four main types, as seen in Figure 1. DESs that are formed from anhydrous metal halide and quaternary ammonium salts are classified as type I [29]. The range of non-hydrated metal halides that have a well point that is suitably low enough to form type I DESs is limited. Consequently, hydrated metal halides were then used to form Type II DESs by virtue of the relatively low cost of many hydrated metal salts coupled with their inherent air/moisture insensitivity. Moreover, Type III eutectics are formed from HBA (quaternary ammonium salts) and HBD (including amides, carboxylic acids, and alcohols) [30,31]. Type III eutectics are easy to prepare and are relatively unreactive with water; many are biodegradable and are of relatively low cost. The wide range of HBDs that are available means that this class of DESs is particularly adaptable. The physical properties of the liquid are dependent upon the hydrogen bond donor and can be easily tailored for specific applications. Finally, type IV DESs consist of metal halides and HBD (amide, alcohol, acids,…etc.) [32]. Type III DESs are the most adaptable type in Analytical Chemistry (especially in DLLME-SFOD).

## 4. DESs in DLLME-SFOD

In the past few years, great progress has been achieved in DES synthesis, physicochemical properties analysis, and structural characterization. Furthermore, the application of DESs in the extraction of chemicals has increased. DLLME-SFOD are among some of the more appealing approaches where DESs have been found to be applicable. In DLLME-SFOD, there is a need for at least two organic solvents (extracting and dispersive solvents). Each type of solvent has certain requirements, and DESs could replace both in various research applications. A detailed explanation of DESs being used as either dispersive or extracting solvents is given in the following sections.

### 4.1. DES as a Novel Disperser in DLLME-SFOD

DLLME-SFOD has various restrictions, most of which are due to the requirement of having a disperser solvent. To improve analyte extraction efficiency, the dispersive solvent should be miscible with both the organic solvent and the aqueous sample in order to efficiently disperse the extracting solvent in water. Changing the disperser has been found to influence extraction efficiency; therefore, it should be considered during optimization. Not only the disperser type but also the disperser volume could affect the extraction efficiency. Lower disperser volumes may not be enough to disperse the extractant well and could result in the formation of a cloudy solution. On the other side, higher disperser volumes may result in the analytes having increased solubility in water due to co-solvency, thus decreasing the extraction efficiency. Short chain alcohol (MeOH, EtOH, propanol), acetone, and acetonitrile are commonly used dispersers. The high impact of this hazardous solvent on the environment has attracted attention and has increased the interest in greener dispersers to replace those hazardous organic ones.

DESs have recently attracted attention as being a green alternative to hazardous conventional dispersers. DESs could serve as dispersers in in DLLME-SFOD by enhancing the dispersion of the extracting solvent in an aqueous sample, thus accelerating the extraction kinetics and enhancing the extraction efficiency. This can be attributed to the salting-out effect (causing by quaternary ammonium salts), the source of protons (generated by carboxylic acids), and the ion-pair agent (formed by halides) for analyte complex formation and extraction.

First, El-Deen et al. reported the use of DES (TBABr: acetic acid, 1:2) as a green disperser in DLLME-SFOD that was used for the enrichment of nine steroids from water samples for the first time [33]. In this procedure, unlike the usual DLLME procedure, the aqueous sample (spiked with the analytes) was quickly injected into the extraction mixture (extracting and dispersive solvents) using a syringe. This could be attributed to the high viscosity of the extraction mixture, which would have hindered their aspiration into the syringe. The cloudy solution that formed as a result was centrifuged to separate the two phases, and it was then transferred to an ice bath in order to allow the organic upper layer to solidify. Finally, the solidified alcohol layer was collected with a spatula and was allowed to melt. It was then subjected to final HPLC analysis. The disperser DES exhibited excellent extraction efficiency compared to the conventional organic disperser. The DES, with its individual components, plays a vital role during the dispersion process through the decomposition of the DES, resulting in the dispersion of the extracting solvent and thus improving the extraction efficiency of the analytes. In addition, the HBA in the prepared DES (TBABr) contains a bromide ion that can act as salting-out agent and that can accelerate mass transfer between the two phases [34]. After that, different articles reported the use of the same DES (TBABr: acetic acid, 1:2) as a disperser in DLLME-SFOD for enriching different chemicals from different matrices. It was used for DLLME-SFOD in order to enrich the preservatives in beverages [35] with the aid of vortexing for 3 min. The method exhibited high EFs (EF = 81–99). Furthermore, it was reported to be successful for the extraction of Co (II) and Ni (II) from water and food samples [36] with the help of ultrasonication for 1 min. The method has a low limit of detection (LOD, 0.8–1.3 µg/L). Finally, another natural DES (NADES) was reported in DLLME-SFOD as being able to disperse pesticides from water and white wine with the aid of vigorous shaking. The NADES consists of lactic acid: glucose: water at a molar ratio of 5:1:3 [37]. The addition of water has a significant effect in decreasing the viscosity of the NADES. The dispersive NADES provided recoveries that were higher than 90% for all of the studied analytes. This could be due to the lower viscosity and higher polarity of the dispersive NADES, which is essential for improving the interaction yield between the aqueous sample, the NADES, and the extracting solvent. Table 1 summarizes the DLLME-SFOD methods using DES as a dispersive solvent.

### 4.2. DES as an Extracting Solvent in DLLME-SFOD

In DLLME, selecting the optimal extracting solvent is critical for increasing the extraction efficiency. Even tiny changes in the solvent’s chemical structure could have an impact on the extraction process. Several conditions must be met by the extracting solvent. To begin with, it must be immiscible with water in order to enable phase separation and analyte partitioning. It should also have a high partition coefficient to guarantee preferential dispersion in the organic droplet. When the disperser is added, it should likewise be dispersible. This step creates a cloudy solution, which significantly increases the contact surface between the aqueous solution and the organic extracting solvent. As a result, some common organic solvents, such as *n*-hexadecane, can be avoided since it is immiscible with common dispersive solvents. Furthermore, it must have a lower density than water to be able to float on the surface, facilitating the separation of the solidified droplet. As a result, halogenated hydrocarbons including carbon tetrachloride, chloroform, and methylene chloride are incompatible with DLLME-SFOD. Moreover, it should have low volatility in order to reduce solvent loss due to evaporation. The quantity of the organic solvent must be kept consistent, or the balance will be disrupted. To achieve preferential dispersion in the organic droplet, it should also have a high partition coefficient. Most crucially, it must have a melting point that is lower than room temperature (between 0–20 °C), allowing the freezing phase to be accomplished with a simple ice bath or through refrigerator and allowing the frozen droplet to melt at ambient temperature following separation from the extraction medium. Most low-density organic solvents utilized in LLE, such as toluene, benzene, and amyl acetate, do not meet this criterion. The solvent should be compatible with instrumental procedures. DLLME-SFOD is only a sample preparation step, and the preconcentrated analytes are further examined using an appropriate instrument. If the solvent is incompatible with the analytical procedure, then it must first be evaporated, which may limit solvent selection. In such case, the additional evaporation step will be labor and time intensive, complicating the extraction operations. Finally, it should be inexpensive and widely available for the procedure to be cost effective. High number of DESs have been found to meet all of those requirements and were hence applied as extracting solvents in DLLME-SFOD for different analytes from various samples.

The volume of the extracting solvent must also be optimized. An increase in the volume of the extracting solvent corresponds to an increase in the amount of extracted analytes, which, in turn, increases the % recovery [38]. This increase in % recovery, however, is deceptive since the enrichment factor (EF) will drop. The drop in EF with increasing volume of the extracting solvent is due to the dilution effect, independent of the amount of analyte that is extracted. It is worth noting that in traditional extraction, the % recovery is an essential metric because the main goal is to extract as much analyte as feasible. The extracting solvent is then evaporated, and the residue is reconstituted in an appropriate amount to form a solution with a high analyte concentration. The volume of the extracting solvent is unimportant in this case. However, the EF is more significant in DLLME-SFOD since the extracted analyte is frequently introduced into the extracting solvent without evaporation. As a result, lower amounts of the extracting solvent are preferred in DLLME-SFOD.

On the other hand, when the partition coefficient is very modest (0.5), the influence of the solvent volume on EF is insignificant. The volume of the extracting solvent is typically in the microliter range [39]. Using a lower volume boosts the EF. As a result, the amount of the extracting solvent should be carefully tuned to increase the extraction efficiency without reducing the volume that is accessible for analysis.

**Figure 2:**
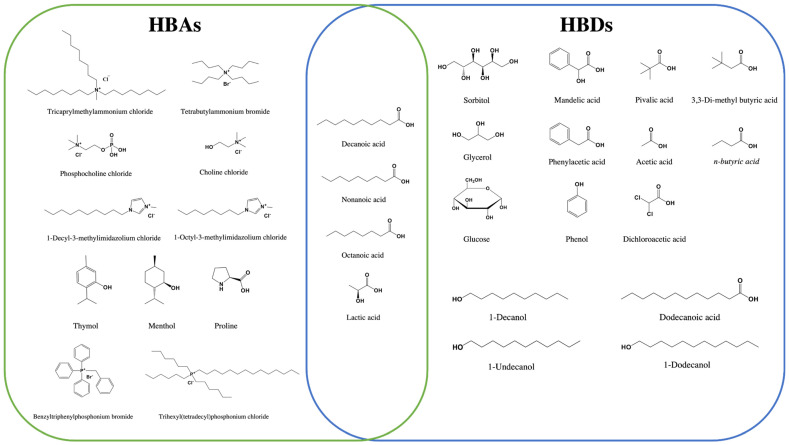
List of the DES components (HBDs and HBAs) used in DLLME–SFOD.

Figure 2 summarizes the components (HBDs and HBAs) that are commonly used in the preparation of DESs for DLLME-SFOD. Those methods are discussed in the next sections. They are classified into methods for organic analytes, and others for inorganic ones.

#### 4.2.1. DES for Extracting Organic Analytes from Different Matrices

The analyte separation efficiency using DES-based DLLME-SFOD prior to instrument detection is dependent on some factors. One important factor is the viscosity of the DES. The higher the viscosity of the DES, the longer the emulsification time, which extends the duration of contact between the DES aggregates and the analytes before phase separation. Thus, DESs with a low viscosity are preferred. However, DES viscosity can also be affected by the branched chain structure and then extraction temperature.

Yang et al. found that a change in the molar ratio of HBD: HBA greatly affects the DES viscosity [40]. DES ([N8,8,8,1]Cl: 1-dodecanol, 1:1) was used as the extracting solvent for the preconcentration of benzoylureas from water samples prior to HPLC analysis. Good recoveries in the range of 82–93% with high precision (%RSD < 5%) and high EFs for the analytes (91–97) were achieved [40]. Furthermore, the viscosity of the DESs decreased as the quaternary ammonium chain decreased. Zeng et al. found that changing the quaternary salt in the DES to [OMIM]Cl, could decrease the DES viscosity, which, in turn, affected the extraction efficiency [41]. The used DES consists of [OMIM]Cl and 1-dodecanol. The analyte recoveries were comparable to those of the previously reported method. However, this method showed higher EFs (171–188) [41].

The extraction efficiency relies heavily on whether there are favorable interactions, such as hydrogen bonding and *p–p*, between the DESs and the analytes. In this sense, the physicochemical properties of the DESs and the analytes are the key factors that dramatically affect the extraction performance. However, the physicochemical properties of DESs are strongly related to the chemical structure and molar ratio of HBA and HBD in DESs. Specifically, the DES polarity has the most significant influence on the solubility between DESs and analytes and is based on the “like dissolves like” principle. The commonly used DESs in DLLME-SFOD are hydrophobic solvents. The formation of hydrogen bonds and/or *p–p* interaction between the hydrophobic DESs (HDESs) and the analytes results in a large decrease in the analyte concentration in the aqueous phase, thereby achieving its separation from water. A HDES (TBACl: decanoic acid) has been used as an extracting solvent in DLLME-SFOD for the simultaneous preconcentration of active curcuminoids in *Curcumae Longae Rhizoma* and in turmeric tea [42]. The method was able to attain EFs that were in the range from 608 to 848 with satisfactory accuracy (84–116%) and precision (%RSD < 4). DESs consisting of TBABr and carboxylic acids were also used for PAH extraction preceding HPLC-FLD analysis [43]. The method exhibited acceptable recoveries of 83–117% and also demonstrated high precision (%RSD < 10%). HDESs were also reported in the DLLME-SFOD for the extraction of aromatic amines [44] and OPFRs [16] from aqueous samples.

On the other hand, the DES viscosity decreases as the temperature increases, which could be demonstrated as Arrhenius-like behavior. Therefore, some DLLME-SFOD were carried out by heating the samples only mildly when viscous DESs were used as the extractant. Amoxicillin and ceftriaxone were found to be present in hospital sewage [45] with the aid of heating at 55 °C in a water bath.

The in situ preparation of DESs has also been reported and aims to reduce the time that is needed for the sample preparation step, thus meeting another green analytical chemistry (GAC) principle. The DES (choline chloride and decanoic acid) was able to be prepared in situ in a milk sample and was able to be simultaneously used to extract pesticides along with the precipitation of milk proteins [46]. Choline chloride was also used with *n*-butyric acid for the in situ formation of DES in edible oil to determine the phytosterol content. The method exhibited high EFs (312–375) with a %RSD < 8% [47].

To reduce the consumption of too many reagents during DES preparation, Shishov et al. and his group reported a novel method based on the in situ formation of DESs through the reaction of the targeted analytes (as HBD) with menthol (as HBA). Mixing the aqueous sample phase (spiked with the targeted NSAIDs) with molten menthol resulted in DES formation and analyte extraction followed by organic phase (extract) separation based on its solidification [48]. The solidified organic phase was then allowed to melt before analysis by means of UPLC-MS/MS.

The DESs that consisted of either quaternary ammonium or phosphonium salts (as HBA) with straight-chain monobasic acids or alcohols (as HBD) were found to be useful in applications determining aromatic amines from water [49], antibiotic residue from sausage [50], pesticides from tomato juice [51], antibiotic residues from hamburger and cow liver [52], and organophosphorus pesticides from edible oil [53]. However, most of those quaternary salts still demonstrate a small amount of toxicity. Therefore, researchers have focused their attention on finding greener alternatives to those HBAs. Terpenes have been used in DES preparation to either replace the quaternary salts (HBA) or to combine them with each other (act as both HBA and HBD). The first terpene that was used was menthol. Mohebbi et al. [54] reported the use of menthol in combination with decanoic acid at a molar ratio 1:2 for the preparation of NADES. The efficiency of the prepared DES was evaluated for extracting antidepressants prior to GC-MS analysis with EFs, and extraction recoveries of 122–147 and 74–89% were obtained, respectively. On the other hand, changing the molar ratio of HBA: HBD could affect the physical characteristics of the prepared DES and could thus affect the extraction efficiency. Menthol was also combined with decanoic acid, but a molar ratio of 1:1 instead. The prepared DES was used to extract fungicides from fruit juice and tea drinks with the help of ultrasonication for 9 min to disperse the DES and to enhance the extraction efficiency [55]. In the same way, menthol was then combined with phenylacetic acid, and the formed DES was used for the DLLME-SFOD of pesticides found in farmer urine and plasma [56] or saliva and exhaled breath condensate samples [57] prior to GC-MS analysis. EFs ranging from 379 to 485 were obtained in urine and from 158–194 in plasma.

Not only the molar ratio but also the type of HBD has a significant effect on the prepared DES and thus on the extraction efficiency. Acids could be also replaced by long chain alcohol (HBA), which was the case for the DES that was prepared by Liu et al. (menthol: undecanol). It was used to extract bisphenols from canned fruit prior to UPLC-MS/MS [58]. Acceptable recoveries ranged from 79–101%, with %RSD < 5% being achieved. Rather than the HBD, the HBA could also be changed. A DES consisting of thymol and octanoic acid was used for the preconcentration of strobilurin fungicides in water, juice, wine, and vinegar samples by HPLC [59], and high extraction recovery was observed (77–107%). Other NADESs were applied for the extraction of different analytes, including patulin in fruit juice and dried fruit samples using the spectrophotometric method [60]; phthalic acid esters from common infusions and soft drinks [61]; endocrine-disrupting compounds (EDCs) in injection solutions and sewage [62]; pyrethroids in cereal samples [63]; bisphenols and PAHs from tea infusions [64]; and benzophenone-UV filters (BP-UV filters) from water samples [65].

Furthermore, the extracting DES in DLLME-SFOD should be stable when it is in contact with water. Surface tension determines the suitability of DESs in interfacial processes in which mass transfer occurs. Higher surface tension values facilitate higher extraction efficiencies being obtained. The interactions between HBA and HBD have a profound effect on the surface tension of DESs. The higher interactions between HBA and HBD provide higher DES surface tension and vice versa. However, DES instability was utilized by Aynaz et al. for the extraction of five pyrethroid insecticides from milk samples prior to their analysis by using GC-FID [66]. He found that the DES (menthol: *p*-aminophenol) decomposed to its components during the dispersion (contact with water) and that menthol formed throughout the solution as tiny droplets. The released menthol acted as a extracting solvent and was able to efficiently enrich the target analytes with good recoveries rates (72–84%), high EFs (257–299), and acceptable repeatability (%RSD ≤ 6.4%). Table 2 summarizes the application of DES in DLLME-SFOD for extracting the organic analytes.

#### 4.2.2. DES for Extracting Inorganic Analytes from Various Matrices

DESs have been found to be suitable for applications for the preconcentration of inorganic analytes using DLLME-SFOD. However, they have fewer applications than those for organic chemicals.

Lead is the only metal from column 14 in the Periodic Table of Elements that has been studied [17,74,75]. Arsenic and selenium are metalloids that have been extracted using DLLME-SFOD [76,77,78,79]. In regard to the transition metals, zinc [80], cobalt [55], copper [17,74], mercury [74,75,77], nickel [55], cadmium [17,74,75,80], and chromium [81] have all been quantified using DES in DLLME-SFOD. Most of the reported methods have been established to determine the total metal concentration; however, only a handful of them involve speciation investigations [56,57,58]. Subsequently, the speciation of arsenic and selenium has been accomplished by subjecting the sample to chemical processes to modify the analyte’s oxidation state [77]. A neutral form is necessary for the extraction of inorganic ions into an extractant DES. As a result, almost all of the DLLME-SFOD applications for metals rely on the creation of hydrophobic chelates. Several applications deal with the identification of inorganic substances in various oxidation states. When a complexing agent reacts with just one form of an inorganic analyte, this may be used for speciation, and the microextraction must be performed in two sample solutions where the metal ion has distinct oxidation states. As a result, oxidation or reduction procedures must be incorporated into the sample pre-treatment. As a result of this, a potassium iodide and sodium thiosulfate mixture was used to reduce As (V) [77], hydrochloric acid was used to reduce Se (VI) to Se (IV) [78,79], and ultraviolet (UV) light and microwaves were used to rapidly convert R-Hg to Hg^2+^ [78]. The total concentration of the analyte was then determined from the treated sample aliquot, and the difference regarding the non-treated aliquot was used to determine speciation.

Firstly, Reza et al. reported a green simple HDES (choline chloride: decanoic acid, 1:2) for the extraction of arsenic, selenium, and mercury from real blood samples [77]. The targeted ions were first complexed with diethyldithiophosphoric acid (DDTP) before being extracted into the DES prior to their determination by iridium-modified tube electrothermal atomic absorption spectrometry (ETAAS). Furthermore, Mostafavi et al. also reported another DES (benzyltriphenylphosphonium bromide (BTPPB) and 1-undecanol) for the extraction of selenium from aqueous samples prior to UV–Vis spectrophotometric analysis [79]. Selenium was complexed with diaminobenzidine hydrochloride (DAB) before being extracted to the DES. The centrifugation step (for phase separation) was eliminated by applying the salting-out effect using NaCl. The method exhibited satisfactory recovery (95–105%).

The electrostatic interactions between heavy metals and quaternary ammonium ions in DESs are very important factors for enriching heavy metal ions from the aqueous sample to the DES phase. The hydrated heavy metal anions can substitute halides in DESs, which results in the formation of a new hydrogen bond between hydrolyzed heavy metal anions and quaternary ammonium ions and the strength of this hydrogen bond being enhanced. DES-DLLME-SFOD was applied for the extraction of heavy metals (Pb, Cd, Hg) from soil and vegetables that had been irrigated with treated municipal wastewater [75]. The heavy metals were first chelated with DDTP before being extracted to the DES (imidazolium chloride ionic liquids and 1-undecanol) prior to their analysis by graphite furnace atomic absorption spectrometry (GFAAS). High EFs (up to 1142) were achieved. DES-DLLME-SFOD was also applied for the extraction of Pb, Cd, Cu, As, and Hg from tea [76], Cr (VI) from urine samples [81], and nickel and cobalt from food and water [55]. The results are summarized in Table 3.

### 4.3. Ternary Deep Eutectic Solvents (TDESs) in DLLME-SFOD

The physical characters of the prepared DESs (mainly density, melting point, and viscosity) play a vital role in the microextraction process. Consequently, controlling those parameters had a significant effect. Researchers have tried to add a third component to the classical two-component eutectic solvents and to evaluate the change in the physical parameters on the extraction efficiency. TDESs were applied to extract different organic and inorganic contaminants. El-Deen et al. [69] introduced novel ternary DESs as extracting solvents for the determination of EDCs from water. The eutectic solvents were prepared by combining various fatty acids that were able to act as both HBAs and HBDs concurrently. Ternary eutectic solvents provided excellent extraction efficiency compared to the corresponding binary eutectic solvents. A preconcentration of up to 134-Fold was achieved and was able to obtain excellent recoveries (90–104 %) and results uncertainty <20%. Another TDES that was formed by mixing sorbitol, menthol, and mandelic acid at a suitable mole ratio was evaluated for its efficiency to extract Cd (II) and Zn (II) ions in aqueous samples prior to their determination by GFAAS [80]. TDESs could be used as both a chelating agent and an extracting solvent. The proposed method was successfully applied in the determination of Cd (II) and Zn (II) ions in water and fruit juice samples. The same solvent was also reported for the determination of Cd (II), Cu (II), and Pb (II) ions in milk samples prior to their determination by GFAAS [17]. The prepared TDES was used as both an extracting and complexing agent in the two reports.

### 4.4. Combination of DES-DLLME-SFOD with Other Sample Treatment Techniques

Because of the simplicity of this matrix, most of the extraction techniques that were based on DLLME-SFOD have been used for the examination of aqueous samples (mostly water). However, due to the interaction of the matrix components with organic solvents, it is more difficult to create a floating droplet that is suitable for injection in instruments for more complicated samples, and therefore, sample pre-treatment is required. DLLME-SFOD was used in conjunction with various pretreatment procedures to identify different analytes in food samples.

DLLME-SFOD based on the in situ synthesis of DES was combined with a dispersive solid phase extraction (*d*-SPE) method for the extraction of some phytosterols from edible oil samples prior to GC-MS analysis [47]. The analytes were adsorbed onto an octadecylsilane sorbent followed by desorption from the sorbent with ethanol as an elution solvent. The eluent was then diluted with deionized water to obtain a homogenous solution to be subjected to DLLME-SFOD. Then, appropriate amounts of choline chloride and *n*-butyric acid were added to the solution and subjected to different temperatures for DES (extracting solvent) formation and dispersion. The cloudy solution that was obtained was placed into an ice bath to be solidified and was allowed melted at room temperature before it was injected into the separation system. The EFs and extraction recoveries of the analytes ranged from 312 to 375 and 75–90%, respectively. Using the same method, DES-DLLME-SFOD was also combined with *d*-SPE for the extraction of different organophosphorus pesticide residues from edible oil samples before determination by GC-NPD [53]. The analytes were first extracted from the spiked oil by *n* –hexane before being vortexed and centrifuged. The supernatant was then transferred into another tube containing PSA sorbent. After that, the solution was sonicated for 4 min. In this step the analytes were adsorbed onto surface of the sorbent. The adsorbed analytes were eluted with acetone under sonication for 3 min. Finally, the acetone phase was separated from the sorbent by centrifugation and was used in DES-DLLME-SFOD. The EFs and extraction recoveries of the analytes ranged from 170–192 and 68–77%, respectively.

A stir bar sorptive extraction (SBSE) method coupled with DES-DLLME-SFOD was used for the simultaneous derivatization and extraction of some acidic pesticides in tomato samples [51]. A stir bar coated with a thin layer of PSA was prepared first. The PSA coated stir bar was vertically released into the tomato juice sample solution (spiked with the analytes), and then it was moved in the solution with the aid of a U–shaped horseshoe neodymium magnet. When it reached close to the top of the tube, the magnet was removed, and the stir bar moved through the solution under gravity. In this procedure, the analytes adsorbed on the surface of the coated stir bar. After the stir bar had passed through the solution eight times, it was removed from the tube and eluted and was then subjected to DES-DLLME-SFOD. The introduced method indicated high enhancement (1543–3353) and enrichment (2530–2999) factors, low limits of detection (7–14 ng/L) and quantification (23–47 ng/L), good linearity (r^2^ ≥ 0.9982), and satisfactory repeatability (% RSD ≤ 12%).

A homogeneous LLE (HLLE) technique was combined with DES-DLLME-SFOD for the extraction of three antibiotics (oxytetracycline, penicillin G, and tilmicosin) from sausage samples [50]. The method showed low LOD, in the ranges of 1.52–2.73 ng/g, demonstrating an %RSD of less than 8%. Finally, counter current salting-out homogenous LLE (CCSHLLE) combined with DES-DLLME-SFOD was used to extract antibiotics from hamburger and cow liver samples [52] before their quantitative analysis by ion mobility spectrometry (IMS). A newly prepared, choline chloride: pivalic acid DES at the μL-level was used. The method revealed good extraction recoveries (67–90%), high EFs (670–900), satisfactory repeatability (%RSD ≤ 6.2), and low LOD (1.7–2.8 ng/g). The results are summarized in Table 4.

## 5. Challenges and Future Perspectives

Despite its undeniable advantages, DLLME-SFOD has significant drawbacks, including the need for at least two organic solvents, the difficulty of automation, and the slowing of the process caused by the freezing step [13,22]. However, intriguing solutions to similar challenges have been revealed. Dispersers can be replaced by greener deep eutectic solvents [33], surfactants can act as dispersers, or the process can be conducted through several mechanical dispersion methods such as ultrasound [49], vortex [72], air [38], or magnetic stirring. On the other hand, the extensive application of DLLME-SFOD has raised the necessity to fully automate the extraction process. The centrifugation and solidification stages impede this development. Significant efforts have been made to eliminate the centrifugation step and to connect DLLME to the next separation process automatically. Solvent-terminated DLLME (ST-DLLME) is useful for demulsifying a mixture and for avoiding the use of a centrifuge [82]. However, the solidification step is still a barrier for automation. Regardless, it is considered to an important step, especially when small volumes of the extractant are used and when it is difficult to distinguish the interfacial surface between the two phases. However, we may expect an increase in the number of published articles that are devoted to innovative approaches in the automation of microextraction techniques in the near future.

## 6. Conclusions

Green analytical chemistry has encouraged chemists to search for new sample preparation techniques that are able to reduce or even eliminate the adverse environmental impact of chemical approaches. Consequently, DESs have attracted extensive attention and have become popular in many fields, including in sample preparation techniques in chemical analysis by virtue of their unique properties, such as easily preparation, low cost, low volatility, tunable properties, and biocompatibility. DESs have found their application in DLLME-SFOD for the enrichment of trace level chemicals in various matrices for the subsequent determination using analytical instruments. Furthermore, the application of DESs in DLLME-SFOD is considered to be a reliable preconcentration method with high enrichment factors and extraction recoveries. Therefore, DESs highlight the potential of DLLME-SFOD as promising green and sustainable sample preparation methods. Nevertheless, the application of DESs in DLLME-SFOD in analytical chemistry is still in its early developmental stages, and the number of DESs that can meet all of the necessary expectations is still limited, with further efforts being required to explore novel DESs. Furthermore, DESs are currently not widely available, limiting their usage to routine analysis in accredited or industrial laboratories. In summary, given the greenness, tunability, and high selectivity offered by DESs, we can anticipate a further increase in the development of novel DESs and that DESs will continue to be very popular in sample preparation in analytical chemistry.

## Figures and Tables

**Table 1:** Application of deep eutectic solvents as a dispersive solvent in DLLME-SFOD different analytes.

Analysis Method	Analytes	Matrix (Amount)	Extractant (Volume)	Disperser DES (Molar Ratio) (Volume)	Assistant Techniques	EF	LOD (µg/L)	% RSD	Ref
HPLC-PDA	**Steroids** (Triamcinolone acetonide, dexamethasone, testosterone, prednisolone, Cortisone, 1,4-androstadiene-3,17-dione, hydrocortisone acetate, Finasteride, 4-androstane-3,17-dione)	Water (5 mL)	1-Dodecanol (50 μL)	TBABr: acetic acid (1:2), (231 μL)	-	44–112	1.0–9.7	<5	[33]
HPLC-PDA	**Preservatives** (benzoic acid, sorbic acid, Methyl paraben, Ethyl paraben, Propyl paraben, Butyl paraben)	Beverages (4 mL)	1-Decanol (80 µL)	TBABr: acetic acid (1:2) (200 µL)	Vortex (3 min)	81–99	20–50	<5	[35]
HPLC-UV	**Pesticides** (fipronil, fipronil-sulfide, fipronil-sulfone, boscalid)	Water and wine (5 mL)	1-Dodecanol (100 μL)	Lactic acid: glucose: water (5:1:3), (2 mL)	Vigorous shaking (1 min)	NA	0.8–1.3	<15	[37]
GFAAS	**Ni (II)** and **Co (II)**	Water and food (10 mL)	1-Dodecanol (75 μL)	TBABr: acetic acid (1:2), (250 μL)	Ultrasonication (1 min)	100	0.2 and 0.4	<3.5	[36]

**Table 2:** Application of deep eutectic solvents as an extracting solvent in DLLME-SFOD for organic analytes.

Analysis Method	Analytes	Matrix (Amount)	Extractant (Molar Ratio), (Volume)	Disperser (Volume)	Auxiliary Equipment	EF	LOD (µg/L)	% RSD	Ref
HPLC-VWD	**Benzoylureas** (triflumuron, hexaflumuron, flufenoxuron, lufenuron)	Water (8 mL)	[N8,8,8,1]Cl: 1-dodecanol (1:1), (50 μL)	FeCl_3_ ethanol solution (250 μL)	-	91–97	0.11–0.35	<5	[40]
HPLC-VWD	**Benzoylureas** (triflumuron, hexaflumuron, flufenoxuron, lufenuron, diflubenzuron)	Water (8 mL)	[OMIM]Cl: 1-dodecanol (1:2), (40 μL)	Vortex (3 min)	-	171–188	0.09–0.16	<6	[41]
GC-MS	**Aromatic amines** (aniline, *p*-toluidine, *p*-chloroaniline, *p*-anisidine, 4-tert–butyl aniline)	Water (10 mL)	ChCl: *n*-butyric acid (1:2), (65 μL)	Aspiration/dispersion (4 cycles)	-	790–940	0.0018–0.006	≤5.3	[44]
HPLC-FLD	**PAHs** (naphthalene, fluorene, phenanthrene, anthracene, fluoranthene, pyrene)	Water (20 mL)	TBABr: decanoic acid (1:2), (80 µL)	NA	Ultrasonic bath (1 min at 35 °C)	163–198	0.0007–0.0066	<11	[43]
HPLC-UV	**NSAIDs** (ketoprofen, diclofenac)	Human urine (8 mL)	Menthol with the studied NSAIDs	NA	Water bath (50 °C)	27–31	15–44	<5	[48]
HPLC-UV	**Antibiotics** (amoxicillin, ceftriaxone)	Hospital Sewage (10 mL)	[DMIM]Cl: *n*-butanoic acid (1:2), (60 μL)	Vortex (5 min)	Water bath (55 °C)	164–172	0.005–0.100	<5.2	[45]
UPLC-MS/MS	**NSAIDs** (diclofenac, flurbiprofen, ketoprofen, mefenamic acid)	Bovine milk (5 mL)	Menthol with the studied NSAIDs	Vortex (1 min)	Heating to 50 °C	81–102	0.01–0.03 µg/kg	<7	[67]
GC-MS	**Pesticides** (prometryn, diazinon, fenvalerate, fenamiphos–sulfone, fenpropathrin, bifenthrin, terbutryn, bromopropylate, deltamethrin, phosalone)	Urine and plasma (5 mL)	Menthol: phenylacetic acid (3:1), (41 μL)	N_2_ stream flow (2.0 mL/min for 2.5 min)	-	Urine (379–485) Plasma (158–194)	Urine (0.002–0.017) Plasma (0.004–0.036)	NA	[56]
GC-MS	**Pesticides** (diazinon; prometryn; terbutryn; bifenthrin; fenpropathrin; bromopropylate; fenamiphos-sulfone; phosalone; fenvalerate, deltamethrin)	Breath condensate and saliva (5 mL)	Menthol: phenylacetic acid (3:1), (41 μL)	Air stream (FR = 2.0 mL/min, for 2.0 min)	-	79–97	0.002–0.059	<7	[57]
GC-MS	**Antidepressants** (Amitriptyline, nortriptyline, Clomipramine, imipramine)	Urine (5 mL)	Menthol: decanoic acid (1:2), (54 μL)	30% Na_2_SO_4_ (1 mL)	NA	122–147	0.013–0.025	<11	[54]
GC-MS	**OPFRs** (Triphenyl phosphate, tripropyl phosphate, TCP, TBP, TCEP, TCPP, TEHP)	Water (5 mL)	BTPPB: 1-undecanol (1:4), (90 μL)	Aspiration/dispersion (11 times)	NA	119–312	0.002–0.023	<8.7	[16]
HPLC-UV	**Curcuminoids** (bisdemethoxycurcumin, demethoxycurcumin, curcumin)	*Curcumae Longae Rhizoma* and turmeric tea (0.5 g)	TBACl: decanoic acid (1:1), (70 μL)	Magnetic stirring at 40 °C	NA	608–848	0.07–0.09	<4.2	[42]
HPLC-UV	**Aromatic amines** (2-chloroaniline, 4-chloroaniline, 1-naphthylamine)	Water (15 mL)	[P_14,6,6,6_]Cl: decanol (1:2), (40 µL)	Ultrasound (60 s)	NA	116–121	0.07–0.11	<6.2	[49]
UPLC-MS/MS	**Bisphenols** (bisphenol S, bisphenol A, bisphenol B)	Canned fruit (0.5 g)	Menthol: undecanol (1:2), (300 μL)	Acetonitrile (400 μL)	Vigorous shaking (2 min)	4.4–4.9	0.0015–0.003 µg/g	<4.6	[58]
GC–FID	**Pesticides** (carbaryl, hexythiazox, pretilachlor, iprodione, famoxadone, sethoxydim, fenazaquin)	Milk (5 mL)	ChCl: decanoic acid (1:2), (63 μL)	Vortex (1 min)	Ultrasonic bath (7 min at 50 °C)	320–445	0.90–3.9	<7	[46]
HPLC-PDA	**Strobilurin fungicides** (Picoxystrobin, pyraclostrobin, trifloxystrobin)	Water, juice, wine, vinegar (10 mL)	Thymol: octanoic acid (1:5), (120 μL)	Effervescence tablet [Na_2_CO_3_ (10 mg), citric acid (80 mg)]	NA	NA	0.15–0.38	NA	[59]
HPLC-PDA	**Benzophenone and UV filters** (BP, BP-1, BP-3, PS, BS)	Water (5 mL)	Decanoic acid: dodecanoic acid (2:1) (65 μL)	Aspiration/dispersion (6 cycles)	NA	41–50	0.045–0.54	≤4.2	[68]
HPLC-PDA	**EDCs** (BPA, BP, EE, DEST, 4-NP)	Water (5 mL)	Nonanoic acid: decanoic acid: dodecanoic acid (1:1:1), (200 µL)	Aspiration/dispersion (6 cycles)	-	38–134	0.96–2.3	<7	[69]
HPLC-UV	**Fungicides** (azoxystrobin, fludioxonil, epoxiconazole, cyprodinil, prochloraz)	Fruit juices and tea drinks (5 mL)	Menthol: decanoic acid (1:1), (70 μL)	NA	Ultrasonication (9 min)	NA	0.75–8.45	<14.8	[70]
UV–Vis	**Patulin**	Fruit juice and dried fruit (2 mL)	L-proline: glycerol (3:1), (410 μL)	Aspiration/dispersion (6 cycles)	NA	150	3.5	<5.6	[60]
HPLC-UV	**Phthalic acid esters** (DPP, BPP, DBP, DCHP, DEHP, DINP, DIDP, diisopentyl phthalate, di-*n*-pentyl phthalate)	Teas Infusion (15 mL) and soft drinks (20 mL)	Menthol: acetic acid (1:1), (100 µL)	NA	Manual vigorous shaking (1 min)	3–12	NA	1–22	[61]
GC-MS	**EDCs** (DEP, DBP, DEHP, BPA, DEHA)	Polyethylene packed injection solutions (5 mL)	Menthol: decanoic acid (1:2), (65 μL)	Aspiration/dispersion (4 cycles)	NA	395–470	0.014–0.033	<7	[71]
HPLC-FLD	**Endocrine disrupting compounds** (estradiol, estriol, BPA, BPF)	Sewage	Octanoic acid: 1-dodecanol (1:3), (80 μL)	Vortex (1 min)	NA	96–111	0.00133–0.00292	<6.2	[62]
HPLC-PDA	**Pyrethroids** (bifenthrin, β-cypermethrin, deltamethrin)	Corn, wheat, barley, oats Cereals (1 g)	Thymol: octanoic acid (1:4), (60 μL)	Acetonitrile (1.5 mL)	NA	NA	2–2.7 μg/kg	<3.6	[63]
HPLC-UV	**Bisphenols and PAHs** (BPF, BPA, BPB, naphthalene, biphenyl, fluorene, phenanthrene, anthracene)	Tea infusions (5 mL)	Menthol: dodecanoic acid (3:1), (100 μL)	Aspiration/ dispersion (9 cycles)	NA	15–18	0.16–0.75	≤2.3	[64]
HPLC-PDA	**Benzoic acid and sorbic acid**	Ketchup and powder bags of instant noodles (10 mL)	Menthol: *p*-aminophenol (1:2), (800 μL)	Vortex (2.6 min)	NA	NA	30 and 80	<5.6	[72]
GC-FID	**Pyrethroid insecticides** (deltamethrin, cypermethrin, bifenthrin, cyhalothrin, permethrin)	Milk (10 mL)	Menthol: *p*-aminophenol (1:2), (94 μL)	Ammonia solution (300 μL)	NA	257–299	1.1–2.4	≤6.4	[66]
HPLC-UV	**Anthraquinones** (rhein, emodin, chrysophanol, physcion)	Fried Cassiae semen tea infusions (10 mL)	ChCl: octanoic acid (1:2), (100 μL)	CO_2_ (H_2_SO_4_ and Na_2_CO_3_ reaction)	NA	94–104	80–110	<3.3	[73]
HPLC-UV	**Benzophenone-UV filters** (BP-1, BP-2, BP-3, BP-6)	Water (10 mL)	[P_4,4,4,12_]BF_4_: decanoic acid (1:9.4)	CO_2_	NA	34–42	0.60–1.50	<8	[65]

**Table 3:** Application of deep eutectic solvents as an extracting solvent in DLLME-SFOD for inorganic analytes.

Analysis Method	Analytes (Details)	Matrix (Amount)	Extractant (Molar Ratio), (Volume, μL)	Disperser	Assistant Techniques	Chelating Agent (Volume, μL)	EF	LOD (µg/L)	% RSD	Ref
ETAAS	As, Se, Hg (Speciation)	Child blood (10 mL)	ChCl: Decanoic acid (1:2), (60 μL)	Vortex (5 min)	NA	DDTP (15 μL)	98–106	0.015–0.10	≤5.8	[77]
GFAAS	Se (Speciation)	Child blood (5 mL)	ChCl: Decanoic acid (1:2), (60 μL)	Vortex (4 min)	NA	DDTP (15 μL)	112	0.05–5	<3.5	[78]
UV-Visible	Se (Speciation)	Water (5 mL)	BTPPB: 1-undecanol (1:4), (700 μL)	Aspiration/ dispersion (7 cycles)	NA	DAB (0.09 %)	315	0.76	≤8.3	[79]
ETAAS	Cr (VI)	Urine (10 mL)	BTPPB: phenol (128 μL)	Sonication (1 min)	NA	DPC	34	0.002	≤4.7	[81]
GFAAS	Pb, Cd, Hg	Soil and vegetables (1 g)	[DMIM]: 1-undecanol (1:2), (50.0 μL)	Vortex (4 min)	Water bath (at 55 °C)	DDTP (15 μL)	114–172	0.01–0.03 μg/kg	≤7	[75]
GFAAS	Pb, Cd, Cu, As, Hg	Tea (0.5 g)	[DMIM]: *n*-butanoic acid (1:2), (60 μL)	Vortex (4 min)	Water bath (at 50 °C)	DDTP (20 μL)	164–235	0.005–0.10 μg/kg	≤3.5	[74]
GFAAS	Ni, Co	Food and Water (50 mL)	Menthol: decanoic acid (150 μL)	NA	NA	Br-PADAP (150 μL)	50	0.3–0.4	≤3	[55]
GFAAS	Cd, Zn	Water and fruit juice (5 mL)	Menthol: Sorbitol: Mandelic acid (1:2:1), (125 μL)	Aspiration/dispersion (9 cycles)	NA	DES	23.4–24.8	0.12–0.15	≤4.2	[80]
GFAAS	Cd, Cu, Pb	Milk (5 mL)	Menthol: Sorbitol: Mandelic acid (1:2:1), (100 μL)	Methanol (1.5 mL)	NA	DES	NA	38–0.42	≤4.5	[17]

**Table 4:** Combination of DES- DLLME-SFOD with other sample pretreatment techniques for extracting various analytes.

Analysis Method	Analytes	Sample Preatment	Matrix (Amount)	Extractant (Molar Ratio), (Volume)	Disperser (Volume)	EF	LOD (µg/L)	% RSD	Ref
IMS	**Antibiotic residues** (oxytetracycline, penicillin G, tilmicosin)	HLLE	Sausage (20 g)	PChCl: dichloroacetic acid: dodecanoic acid (1:1:1), (70 µL)	Acetonitrile (2 mL)	1260–1580	0.00152–0.00273 µg/g	<8	[50]
IMS	**Antibiotic residues** (oxytetracycline, penicillin G, tilmicosin)	CCSHLLE	Hamburger and cow liver (10 g)	ChCl: pivalic acid (1:2), (75 μL)	Acetonitrile (1 mL)	670–900	0.0017–0.0028	≤6.2	[52]
GC/MS	**Phytosterols** (brassicasterol, campesterol, stigmasterol, β-sitosterol, lupeol)	*d*-SPE	Edible oil (5 mL)	ChCl: *n*-butyric acid (0.065 g: 80 µL, in situ formation)	Water bath (5 min at 75 °C)	312–375	0.52–1.6	≤8.2	[47]
GC-MS	**Pesticides** (Dalapon, 2-methyl-4-chlorophenoxyacetic acid, 2,4-dichlorophenoxyacetic acid, fenoxaprop, haloxyfop)	SBSE	Tomato juice (50 mL)	ChCl: *n*-butyric acid (1:2), (58 μL)	NA	2530–2999	0.007–0.014	≤12	[51]
GC-NPD	**Organophosphorous pesticides** (Etrimfos, fenthion, di-azinon, chloropyrifos)	*d*-SPE	Edible oil (2.5 mL)	ChCl: 3,3-dimethylbutyric acid (1:1), (15 µL)	NA	170–192	0.06–0.24	≤9.2	[53]

## Data Availability

Not available.

## Sample Availability

Samples of the compounds are not available from the authors.

## Abbreviations

**[DMIM]Cl**: 1-decyl-3-methylimidazolium chloride, **[N8,8,8,1]Cl**: tricaprylmethylammonium chloride, **[OMIM]Cl**: 1-octyl-3-methylimidazolium chloride, **[P14,6,6,6]Cl**: trihexyl(tetradecyl)phosphonium chloride, **[P4,4,4,12]BF4**: tributyl(dodecyl)phosphonium tetrafluoroborate, **4-NP**: 4-nonylphenol, **BBP**: butyl benzyl phthalate, **BP**: benzophenone, **BP-1**: 2,4-dihydroxy-benzophenone, BP-2: benzophenone-2, **BP-3**, 2–hydroxy–4–methoxy–benzophenon, **BP-6**: benzophenone-6, **BPA**: bisphenol A, **BPB**: bisphenol B, **BPF**: bisphenol F, **Br-PADAP**: 2-(5-Bromo-2-pyridylazo)-5-(diethylamino) phenol, **BS**: benzyl salicylate, **BTPPB**: benzyltriphenylphosphonium bromide, **BP-UV filters**: benzophenone-UV filters, **CCSHLLE**: counter current salting-out homogenous liquid–liquid extraction, **ChCl**: choline chloride, **DAB**: diaminobenzidine hydrochloride, **DBP**: dibutyl phthalate, **DCHP**: dicyclohexyl phthalate, **DDTP**: diethyldithiophosphoric acid, **DEHA**: di-(2-ethylhexyl) adipate, **DEHP**: di(2-ethylhexyl) phthalate, **DEP**: diethyl phthalate, **DES**: deep eutectic solvent, **DEST**: diethylstilbsterol, **DIDP**: diisodecyl phthalate, **DINP**: diisononyl phthalate, **DLLME-SFOD**: dispersive liquid–liquid microextraction based on solidification of floating organic droplet, **DPC**: 1,5-diphenylcarbazone, **DPP**: dipropyl phthalate, ***d*-SPE**: dispersive solid phase extraction, **EDCs**: endocrine disrupting chemicals, **EE**: ethynylestradiol, **ETAAS**: electrothermal atomic absorption spectrometry, **EF**: enrichment factor, **GAC**: green analytical chemistry, **GC-FID**: gas chromatography-flame ionization detector, **GC-MS**: gas chromatography-mass spectrometry, **GC-NPD**: gas chromatography-nitrogen-phosphorus detector, **GFAAS**: graphite furnace atomic absorption spectrometry, **HBA**: hydron bond acceptor, **HBD**: hydrogen bond donor, **HDESs**: hydrophobic deep eutectic solvents, **HLLE**: homogenous LLE, **HPLC-FLD**: high performance liquid chromatography-fluorescence detector, **HPLC-PDA**: high performance liquid chromatography-photodiode array detector, **HPLC-UV**: high performance liquid chromatography-ultraviolet detector, **HPLC-VWD**: high pressure liquid chromatography-variable wavelength detector, **IMS**: ion mobility spectrometry, **LOD**: limit of detection, **LLE**: liquid–liquid extraction, **NA**: not available, **NADESs**: natural deep eutectic solvent, **NSAIDs**: non-steroidal anti-inflamatory drugs, **OPFRs**: organophosphorus flame retardants, **PAHs**: polycyclic aromatic hydrocarbons, **PhChCl**: phosphocholine chloride, **PS**: phenyl salicylate, **RSD**: relative standard deviation, **SBSE**: stir bar sorptive extraction, **ST-DLLME**: solvent terminated-dispersive liquid–liquid microextraction, **TBABr**: tetrabutylammonium bromide, **TBACl**: tetrabutylammonium chloride, **TBP**: tri-n-butylphosphate, **TCEP**: tri(2-chloroethyl) phosphate, **TCP**: tricresyl phosphate, **TCPP**: tri(2-chloroisopropyl) phosphate, **TDESs**: ternary deep eutectic solvents, **TEHP**: tri(2-ethylhexyl)phosphate, **UPLC-MS/MS**: ultra pressure liquid chromatography-mass spectrometry/mass spectrometry, **UV-Vis**: ultraviolet-visible spectrophotometer.
